# Study and Characterisation of Bimetallic Structure (316LSI and S275JR) Made by Hybrid CMT WAAM Process

**DOI:** 10.3390/ma17225422

**Published:** 2024-11-06

**Authors:** Alejandro Pereira, Antonio Alonso, Primo Hernández, Javier Martínez, David Alvarez, Michal Wieczorowski

**Affiliations:** 1 Faculty of Industrial Engineering, Universidade de Vigo, 36310 Vigo, Spain; primo@uvigo.es; 2 Grupo Precisgal S.L., PTL de Vigo, Calle B 10.06, 36312 Vigo, Spain; antonio.alonso@precisgalgroup.com; 3 Hergome S.L., Estrada Puxeiros—Mos, nº 76/nave 5., 36416 Mos, Spain; hergome@hergome.com; 4 ENCOMAT, CINTECX—Research Center in Technologies, Energy and Industrial Processes, University of Vigo, Lagoas-Marcosende, 36310 Vigo, Spain; davidag@uvigo.gal; 5 Faculty of Mechanical Engineering, Poznan University of Technology, Piotrowo Street 3, 60-965 Poznan, Poland; michal.wieczorowski@put.poznan.pl

**Keywords:** 316LSI, CMT-WAAM, Hybrid-WAAM, metallography, tensile test

## Abstract

The main objective of this research is to conduct an experimental investigation of the bimetallic material formed by 316LSI stainless steel and S275JR structural steel, produced via hybrid wire arc additive manufacturing technology with cool metal transfer welding and machining, and with the objective of being able to reduce the industrial cost of certain requirements for one of the materials. A methodological investigation has been carried out starting with welding beads of 316LSI on S275JR plates, followed by overlapping five beads and conducting final experiments with several vertical layers, with or without intermediate face milling. The results achieved optimal bead conditions for wire speeds of 4 m/min and 5 m/min at a travel speed of 400 mm/min. Overlap experiments show that the best deposition results are obtained with an overlap equal to or greater than 28%. Cooling time does not significantly influence the final geometry of the coatings. Regarding metallographic analysis, the filler material presents an austenitic columnar structure. In the base material, a bainitic structure with inferred grain refinement was detected in the heat-affected zone. An increase in hardness is observed in the heat-affected zone. In the results obtained from the tensile tests of the bimetallic material, an increase in mechanical strength and yield strength is observed in the tested specimens.

## 1. Introduction

Additive manufacturing stands as a key technology within the context of the Fourth Industrial Revolution, capturing notable interest from both researchers and industry stakeholders in recent times [1]. Wire arc additive manufacturing (WAAM) is a technique that offers an economic process with high deposition rates, but with the drawback of poor accuracy [2,3,4], and the requirement of a base substrate on which to deposit the material. Other problems include the lack of dimensional accuracy, poor surface quality, the appearance of porosities that minimize the mechanical properties, or the presence of residual stresses [5,6]. 

The possibility of combining different materials for the manufacture of the same part is currently being investigated. Vinoth et al. [7,8] studied the microstructural properties of AISI316L fabricated by WAAM and concluded that it is a practical and efficient process. The team of Vora et al. [9] used a GMAW-based WAAM technique to fabricate a multilayer structure at optimized process parameters on stainless steel 316L using metal wire of 316L. The tensile test results of the upper, middle, and lower zones developed by the WAAM process are within the range of values for forged 316 stainless steel. Furthermore, the overall results obtained have shown that the structure built by the WAAM process based on GMAW conforms to standards for industrial applications. The results studied by the team of Vu and Thao Le [10] indicate that 316 stainless steel walls made by WAAM show cellular and columnar dendrites at the bottom and equiaxial grains at the top. The tensile strength of the interface region between the material deposited at the bottom is the highest. 

Bimetallic structures [11] can be applied when two different zones have specific property requirements. Bimetallic structures combine metals such as steels, stainless steels, nickel-based steels, aluminum, or titanium, which are used in the aerospace, marine, and automotive industries. The benefit of using bimetallic structures focuses on reducing the cost of the part by using the expensive material only at the specific location where it is needed [12,13,14,15]

Rodrigues et al. [16] investigated and fabricated a functionally graded material from 316L stainless steel to Inconel 625 using different deposition strategies (called direct and soft interfaces) by Twin-Wire and Arc Additive Manufacturing (T-WAAM).

Saha et al. [17] observed that increased heat input leads to a wider weld bead with minimal alterations in penetration and reinforcement. Consequently, it is advisable to employ higher heat input when cladding austenitic stainless steel onto low alloy steel. Murugan et al. [18] have optimised the process parameters, using a genetic algorithm (GA), in order to achieve minimum dilution, maximum reinforcement, minimum penetration, and maximum bead width by optimising the material. 

Cold metal transfer, or CMT, a modified form of gas metal arc welding (GMAW), offers low energy input capabilities [19]. By advancing the wire electrode into the weld pool and retracting it after a certain amount of time, the CMT process mechanically regulates material transfer as well as the beginning and length of short circuits [20].

As defined by CIRP, “Hybrid manufacturing processes are based on simultaneous and controlled interaction of process mechanisms and/or energy sources/tools having a significant effect on the process” [21]. The integration of additive manufacturing and subtractive manufacturing dates back to the late 1980s and early 1990s. Particularly, when considering additive manufacturing as a key element, it is possible to talk about hybrid additive manufacturing, which involves the controlled application of different additive manufacturing processes in combination with traditional manufacturing processes that can occur upstream or downstream [22]. For instance, Chen et al. [23] indicated that hollow cavities must be manufactured by developing several phases of additive manufacturing and machining.

One of the objectives in this initial research was to facilitate cost reduction in the naval, industrial, and biomedical sectors by increasing the corrosion resistance of base carbon steel through coating with stainless steel. 

Specifically in the field of shipbuilding, material coatings for corrosion protection are an important cost factor in the construction and repair of components. If we focus the problem on a propulsion shaft or a rudder stock, the mechanical performance of a carbon steel shaft would imply an equivalent in austenitic stainless steel with a larger section and therefore higher cost, or the use of a complex stainless steel like duplex and similar.

A typical solution for a toughness–corrosion compromise is to encase a steel core with stainless steel but, with the aim of finding an optimized solution to the mentioned problem, the use of these news trends in hybrid manufacturing technologies could be useful.

When focusing on a pair of materials typically used in shipbuilding, such as S275 and AISI 316LSi austenitic stainless steel, the silicon content is explained in terms of preventing chromium reduction due to carbon presence for stainless steel coating.

Several works indicate that additive manufacturing improves the corrosion resistance of SS compared with wrought samples [24,25,26,27]. Higher corrosion resistance is associated with the rapid cooling of AM, which reduces nucleation and crystal growth. This produces a homogeneous distribution of alloying elements, while avoiding the formation of chromium-depleted regions, reducing inclusions such as MnS, and minimizing the nucleation of pits [24,25]. The worst corrosion resistance has been associated with the presence of small amounts of δ-ferrite, microsegregation, and a high level of porosity [26].

The main objective of this project is to carry out an experimental study of the bimetallic material formed by 316LSI stainless steel and S275JR structural steel, produced via hybrid WAAM technology with CMT welding and machining. In this initial step, the research has focused on a dimensional analysis based on different deposition strategies, such as the influence of the manufacturing process on the interface behavior, through mechanical tests, and macro and micro visual inspection of defects and discontinuities. 

## 2. Materials and Methods

All details concerning the experimental procedure are presented. First of all, the research methodology is shown. The materials used are described. The equipment used for the WAAM process is also described. Furthermore, the measurement and test procedures used to evaluate the samples are presented.

### 2.1. Experimental Plan

**Figure 1:**
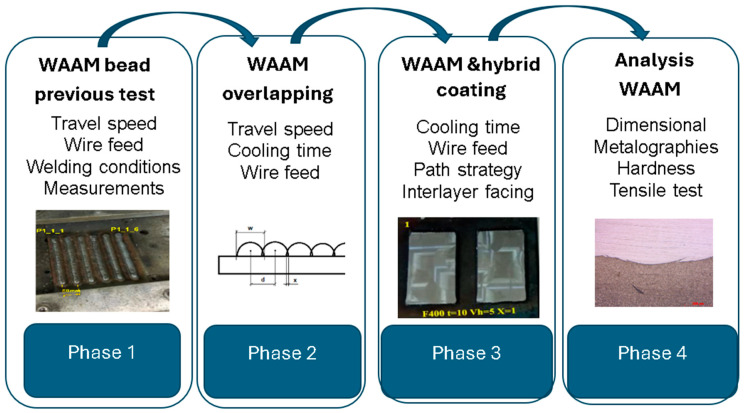
Phases of experimental plan.

The aim of this work is to analyze the coating of 316LSI manufactured by WAAM on S275JR structural steel. The experimental plan was carried out in four phases, which can be seen in Figure 1.

**Figure 2:**
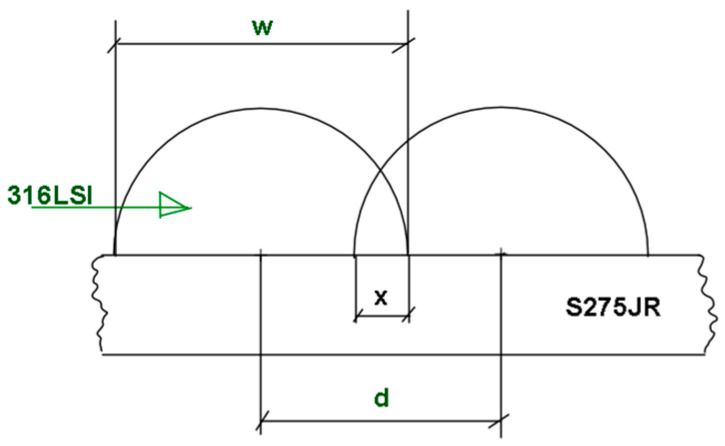
Cladding samples.

- First phase: previous experiments were carried out to choose the most suitable parameters for the weld bead. The selected welding parameters are wire feed (4, 5, and 6 m/min), arc correction (−25, −15, and 0%), and travel feed (from 150 to 500 mm/min). The fixed WAAM parameters were: CMT, two time modes, and argon gas flow rate of 10 l/min. These parameters are significantly different from those used by Huang et al. [28] due to the different targets. In this case, the target was to increase the ratio of deposition without minimal dispersion of bead shapes.
- Second phase: First coatings with parallel beads (C1, C2, and C3 experiments) were obtained with different strategies (zig zag, back and forth), overlap spacing, and cooling time.
  After obtaining these first samples, the manufacturing of the first layer of coatings from five parallel weld beads were carried out. The design of the experiments aims at choosing the best strategy, varying the overlapping (*x* = 0 mm, *x* = 0.5 mm, *x* = 1 mm, and *x* = 1.5 mm, where *x* is shown in Equation (1)), and the cooling time (10s and 40 s). Figure 2 shows the description of the relation between distance (*d*), width of bead (*w*) and overlapping (*x*)

(1) x=w−d

where *x* is the overlapping, *w* is the width of the welding bead, and *d* is the distance between parallel beads.

- Third phase: One first experiment (C4), was conducted with overlapping selected, 15 horizontal beads, and three vertical layers, one on top of the other, with two different vertical welding directions (same direction and cross direction) without machining. The last experiment (C5) was conducted with two vertical layers and two directions (same and cross direction), with face machining between layers. The machining had been conducted using a Microcut milling machine with CNC Fagor 8065 (Buffalo machinery Company Limited, Taichung City, Taiwan).
- Fourth phase: This phase includes dimensional measuring of all the beads, micrographs of selected beads, and selected samples from experiment C5, measuring the hardness of selected beads and conducting a tensile test of selected samples.

### 2.2. Materials

The welding wire material used is AWS A5.9:ER316LSi with a diameter of 1 mm, supplied in 5 kg coils by NickelAlloys S.L (Madrid, Spain). The alloy offers good weldability and corrosion resistance and finds application in pressure vessels for chemicals, food industry, ships, aviation, and biomedicine. The chemical composition of the wire material is obtained via an X-Ray Fluorescense Analyzer Olympus (Precisgal Group Lab, Vigo, Spain), as shown in Table 1.

The second material will act as a substrate and is a structural steel called S275JR according to EN10025-1 [29]. The chemical composition of the substrate material is provided by the supplier, as can be shown in Table 2. Precisgal Group supplied cut plates of S275JR with dimensions of 150 mm × 150 mm × 15 mm.

The shielding gas to be used during the experiments is a mixture of CO_2_ and Argon, in percentages of 2% and 98%, respectively, obtained from AirLiquide, with the commercial name Arcal Chrome Smartop (Al Air Liquide España, s.a., Madrid, Spain).

### 2.3. WAAM Equipment

**Figure 3:**
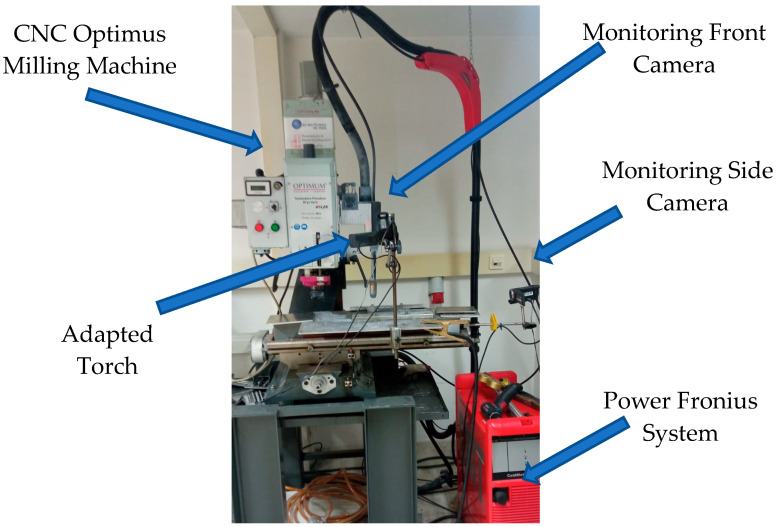
Integration of the CNC Optimus and Fronius CMT systems.

The experimental equipment used in WAAM manufacturing lab was composed of two different systems. The welding machine used was a Fronius TPS 4000 CMT R (Fronius International GmbH, Wels, Austria) machine, which allows welding using Fronius^®^ patented CMT technology. The results are reduced welding temperature and optimized wire movement. Thus, the machine offers a better weld seam quality than the conventional GMAW process. Moreover, the authors integrated the welding machine with the positioning system. The BF 30 Vario Optimum CNC milling machine (Optimum Maschinen Germany GmbH, Hallstadt, Germany) efficiently manages the movement of the entire system. This machine was adapted to position the torch weld along the Z axis. The movement of the X–Y table of the CNC system makes it possible to deposit a layer of weld on a fixed Z level, with the welding torch attached to the milling machine head. An auxiliary worktable was required to electrically isolate both systems. Figure 3 depicts the experimental setup described in this section [5,19,30].

### 2.4. Measurements

#### 2.4.1. Dimensional Measurements

Dimensional measurements were carried out on the welding seams made in previous tests to obtain optimal conditions (smaller dispersions of height and width) and carry out the coating tests.

**Figure 4:**
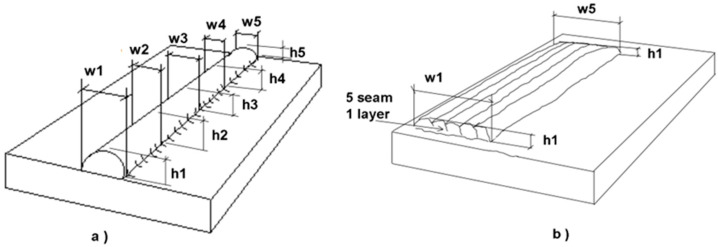
Dimensional measurements: (**a**) welding seams; (**b**) cladding samples.

Figure 4 shows the scheme used for dimensional measuring. A caliper was used to measure the width in five points and another five points to measure the height of the welding beads. The average height and width (*ha*, *wa*), and their respective standard deviation (*hs*, *ws*), were calculated. The same procedure was used to determine the overlay height by measuring at five points on the overlay. The width was measured at five points in the different experiments with 5 and 15 beads of coating width.

#### 2.4.2. Metallography and Hardness

The first step is manual cutting with the Neurtek cutter to section the specimen into different parts. Using the disc saw, the specimens are cut precisely. Afterward, it must be ensured that the surface is completely free of burrs by filing and polishing the surface. Encapsulation is carried out with KEM 35 resin. Once the samples have been encapsulated, they are polished to an average roughness of Ra = 0.8 µm, using a Neurtek^®^ rotary polisher (Neurtek instruments, Eibar, Spain). Subsequently, the sample can be attacked with chemical reagents.

**Figure 5:**
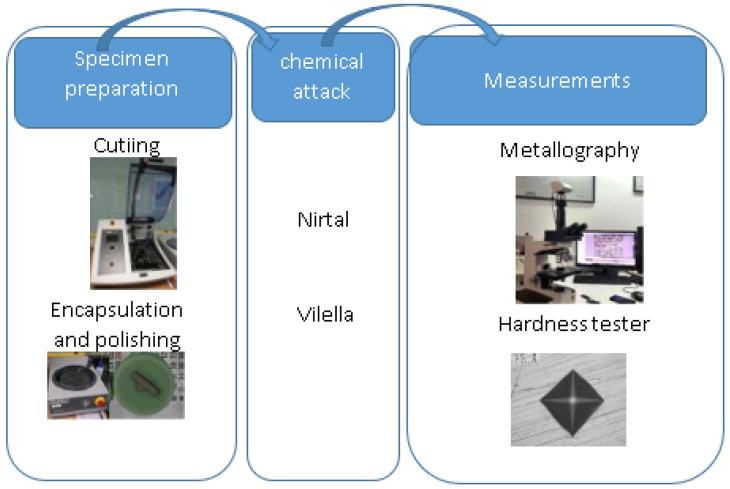
Metallography and hardness procedure.

To reveal the microstructure of the base steel S275JR and stainless steel 316LSI, Nirtal and Vilella are used, respectively. Measurements are made under a Zeiss^®^ microscope (Zeiss Gruppe, Jena, Germany) by capturing the images and recording the measurement conditions. The objective is to analyze the different zones of the weld, the base steel zone, and the 316LSI zone. Figure 5 shows the procedure of the metallography and hardness test.

Finally, microhardness tests, according to Vickers HV0.1, are carried out with the Neurtek^®^ hardness tester (Neurtek instruments, Eibar, Spain) [31] in 21 points of differentiated zones:

- 5 points in the filler material (316LSI), heat-affected zone (HAZ);
- 4 points in the base material, S275JR;
- 12 points measured at 316LSI near HAZ, HAZ, and S275JR near HAZ.

#### 2.4.3. Tensile Test

**Figure 6:**
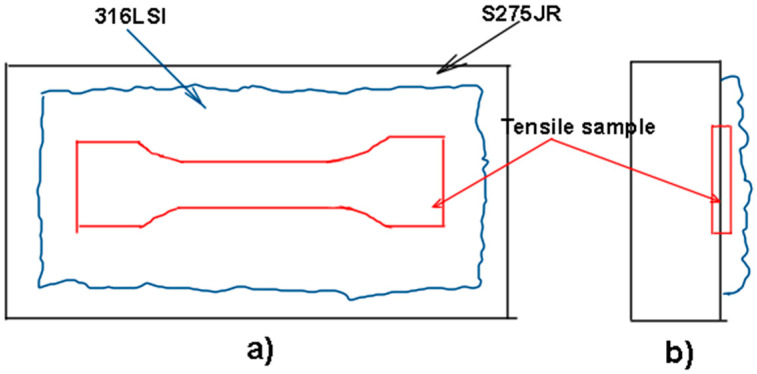
Sketch of machining of tensile samples (**a**) top view (**b**) Left view.

The tensile test was carried out in accordance with ISO 6892-1:2019 [32], using a universal test machine, Shimadzu AG-I (Shimadzu coorporation, Kyoto, Japan) 250 kN. To be able to use this geometry on the welded plates, the dimensions of the selected specimen are scaled to 9:10. The tensile samples must be machined, as can be shown in Figure 6. A specific tool was used to avoid damaging the jaws of the machining center. A test speed of 10 mm/min has been selected.

## 3. Results and Discussion

### 3.1. Dimensional Analysis

According to the methodology, previous experiments were carried out to find the optimum conditions for the 316 bead on the S275JR to improve the coating and avoid defects. Table 3 presents the data from the bead experiments with measurements of the mean width and height and their respective standard deviation. Table 3 also shows the calculation of the flow rate, Equation (2), *Q_s_*, taking into account the wire feed rate, *W_f_*, the travel speed, *T_s_*, and the wire diameter, ER316 (1 mm).

**Table 3:** Data of bead experiments.

Number	Code	*W_f_* (m/min)	*Q_w_* (mm^3^/s)	*T_s_* (mm/min)	*ha* (mm)	*hs* (mm)	*wa* (mm)	*ws* (mm)	*S_b_* (mm^2^)
Test 2	P_1.2.2	4	52.36	200	4.3	0.5	7.0	0.4	15.7
Test 2	P_1.2.3	4	52.36	150	4.5	0.7	7.8	0.7	20.9
Test 2	P_1.2.4	4	52.36	300	3.2	0.8	5.2	0.2	10.5
Test 2	P_1.2.5	4	52.36	400	3.0	0.3	3.8	0.5	7.9
Test 2	P_1.2.6	4	52.36	500	2.7	0.4	3.5	0.5	6.3
Test 5	P_1_5.1	4	52.36	400	2.9	0.1	3.5	0.5	7.9
Test 5	P_1_5.2	5	65.45	400	3.3	0.3	4.3	0.4	9.8

(2) $$Q_{w}=W_{f}\times S_{w}=W_{f}\times (\frac{\pi \times {\emptyset_{w}}^{2}}{4})$$

where:

- $Q_{w}$: Flow rate of material;
- $W_{f}$: Wire feed;
- $S_{w}$: Wire section;
- $\emptyset_{w}$: Wire diameter.

As this flow rate, *Q_w_*, must be like the deposition flow rate, *Q_b_*, the weld bead cross section *S_b_* is obtained, as illustrated in Equation (3).

(3) Qw=Qb →   Sb=QwTs

where:

- $Q_{b}$: Flow rate of bead deposition;
- $S_{b}$: Bead section;
- $T_{s}$: Travel speed.

**Figure 7:**
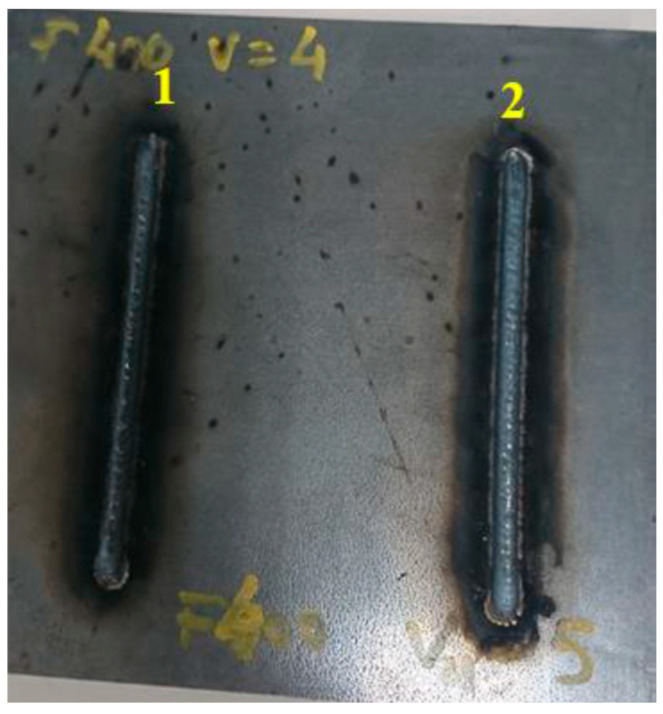
Test 5 (1) bead with *wf* = 4 m/min and *ts* = 400 mm/min, (2) bead with *wf* = 5 m/min and *ts* = 400 mm/min.

As a result of various experiments, it was concluded that the most stable beads were produced at wire speeds of 4 and 5 m/min, and at travel speeds of 400 and 500 mm/min. Figure 7 shows the plate from Test 5, with two beads at 4 and 5 m/min, respectively, and 400 mm/min. This plate was used in Test 5 for hardness measurement.

**Figure 8:**
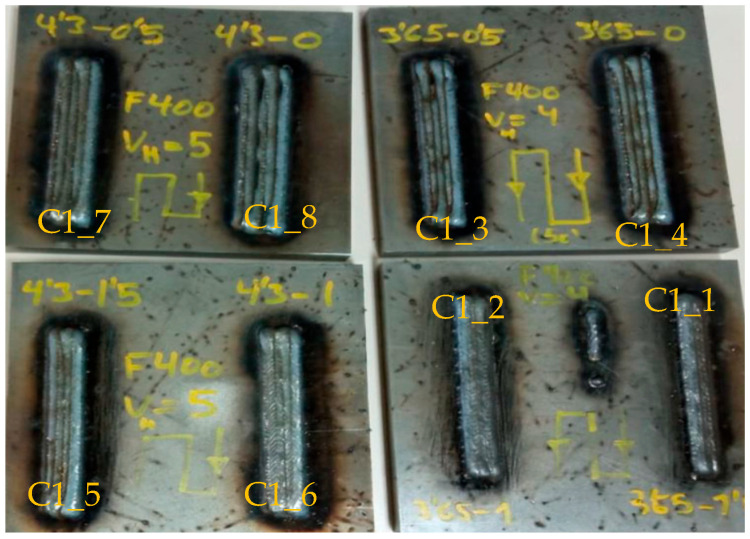
Experiments C1.

Figure 8 shows the specimens obtained in experiment C1, where the defects of samples C1_8, C1_3, and C1_4, consisting of the appearance of intermediate voids, can be seen at a glance.

Table 4 shows the dimensional results of the experiments with codes C1, C2, C3, C4, and C5. The objective of experiments C1, C2, and C3 are performed to find the best conditions for overlapping, i.e., an overlap with five horizontal beads (h_b) is performed. The overlap distance (ov_l), which is subtracted from the bead width distance (*wa*), is varied with a zig-zag strategy (p_st) and a wire feed (*wf*) of 4 and 5 (m/min). The samples C1_4 and C1_8 have an overlap of 0% (ov_1/*wa*) and defects with intermediate voids can clearly be observed. The overlapping of sample C1_3 is 14%, with voids also being observed. Voids disappear with overlaps equal to ov_1 ≥ 1, i.e., above 22%. According to Sahu et al. [33], with a 50% overlap, the metallic particles from the edges of the individual track were mixed with the previous diluted track and formed an almost uniform layer with improved mechanical properties.

It is decided to eliminate the zigzag strategy due to visual failures (Figure 8). Moreover, as this strategy is continuous, it is not possible to determine the cooling time between beads, so it is considered to be equal to 0 s. The cooling time (*ct*) is included in experiments C2 and C3, with 10 s and 40 s of cooling time, respectively.

Experiment C4 includes samples with 15 horizontal beads and up to three layers (v_l), with alternating cross deposition in the second and third layer or in the same direction (cross layer). Finally, experiment C5 includes samples with 15 horizontal beads and two layers, but with intermediate machining (mach) between layers to avoid internal defects. The change of the deposition direction is also included (cross layer).

**Figure 9:**
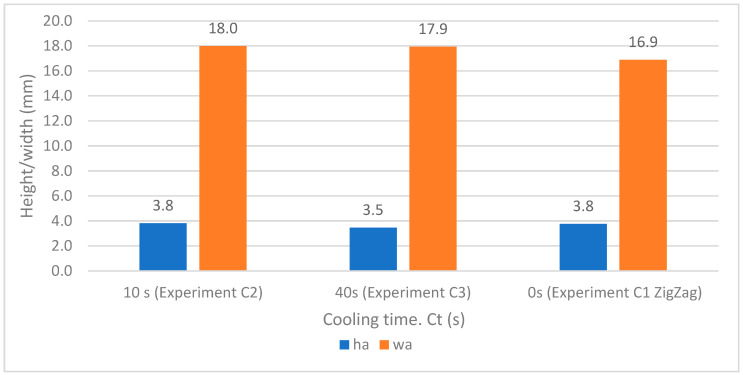
Average height and width versus cooling time.

Figure 9 shows the behavior of the average weld bead heights and widths as a function of the cooling time, considering that in the case of experiment C1 with the zig-zag strategy the deposition is continuous, so the cooling time is zero. For the value of 5 weld bead widths, there is no significant variation with respect to the cooling time; according to Sales et al. [34], the cooling rate changes and differences in the interpass temperature had little effect on mechanical properties. It can, therefore, be stated that the cooling time does not significantly influence the dimensional shape of heights and widths in the coating experiments (C2 and C3). The shortest cooling time is selected as it achieves the highest productivity, and therefore a cooling time of 10 s has been chosen for experiments C4 and C5.

**Figure 10:**
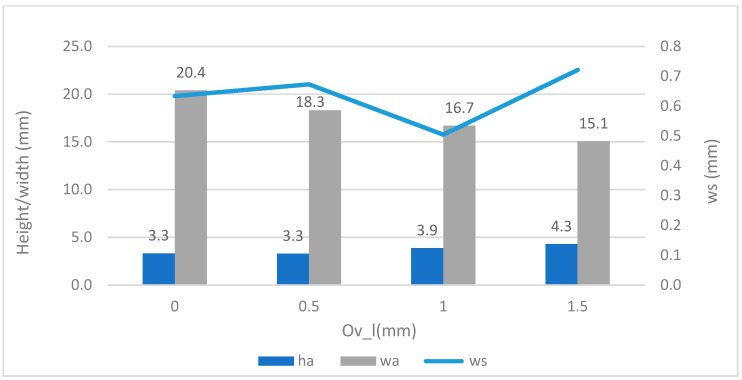
Average height and width vs. overlapping (Ov_l).

Figure 10 shows the average heights (ha) and widths (*wa*), as well as the width dispersion (*ws*) versus the overlap reduction factor (ov_l), in the horizontal weld beads. The highest overlap factor would be 1.5 mm; however, this factor has not been chosen as it has the highest dispersion of 0.7 mm. The overlap selection obtained was the factor ov_l = 1, equivalent to 28%, in experiments C4 and C5, with several vertical layers.

**Figure 11:**
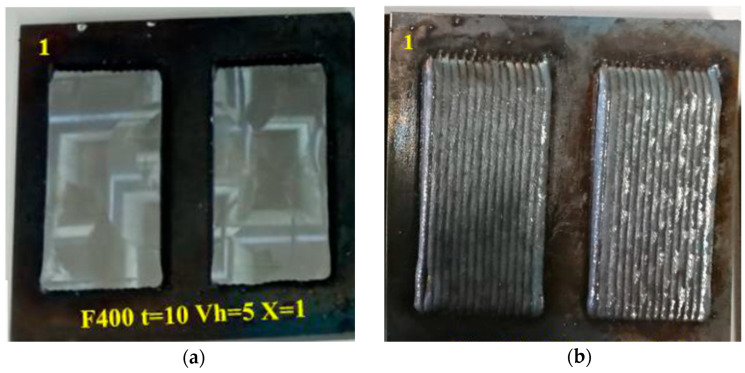

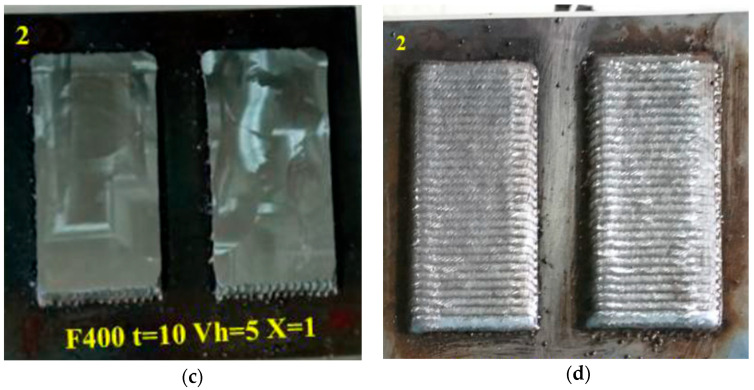
Experiment C5, where the first horizontal layer has been facing: (**a**) Machining of two samples with *ts* = 400 mm/min, *wf* = 5 m/min; (**b**) Second layer of samples C5-5 and C5-6 with same direction; (**c**) Other two samples with *ts* = 400 mm/min, *wf* = 5 m/min; (**d**) Second layer of samples C5-9 and C5-10 with cross layer direction.

Figure 11 shows the specimens obtained from experiment C5, corresponding to a wire speed of 5 mm/min, a travel speed of *ts* = 400 mm/min, a cooling time of 10 s, and overlapping (ov_l = 1). The particularity concerning experiment C4 is that, after giving the first layer with the conditions obtained previously, the specimens are machined to eliminate porosities. Afterwards, the second coating layer is obtained on the machined layer, with differentiation between the same and crossed directions.

**Figure 12:**
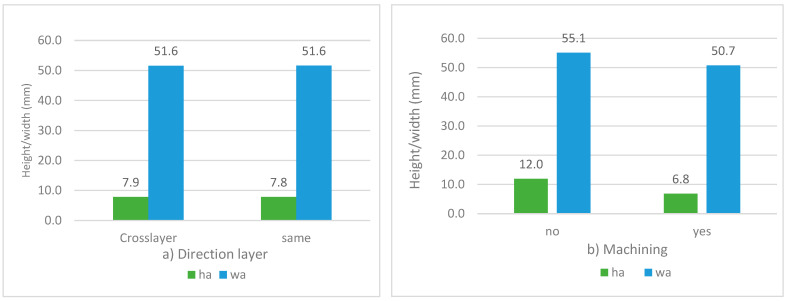
Dimensional results of experiments C4 and C5: (**a**) Average heights and widths versus direction of layers; (**b**) Average heights and widths versus facing.

Figure 12a shows the possible influence of crossing vertical layer directions on the average heights and widths of vertical layer coatings. No significant influence is observed, as the widths are similar and the average heights do not change significantly by changing the layer direction (7.9 and 7.8 mm). As a result, it can be stated that the layout of vertical paths has no influence on the dimensioning of the 316LSI coating.

Figure 12b shows the possible influence of machining the intermediate layer on the average heights and widths of the vertical layer coating. In this case, a greater width is observed in the unmachined coatings, as well as a layer height corresponding to 12 mm, which means a height for each layer of 4 mm/layer without machining. In the case of intermediate machining, the height per layer is assumed for the same *wf* = 5 m/min and *ts* = 400 mm/min, equal to 3.4 mm. These height differences are logical because the face machining was carried out with an axial depth of 2 mm, which reduces the height of the coating.

### 3.2. Metallographs and Hardness Test

**Figure 13:**
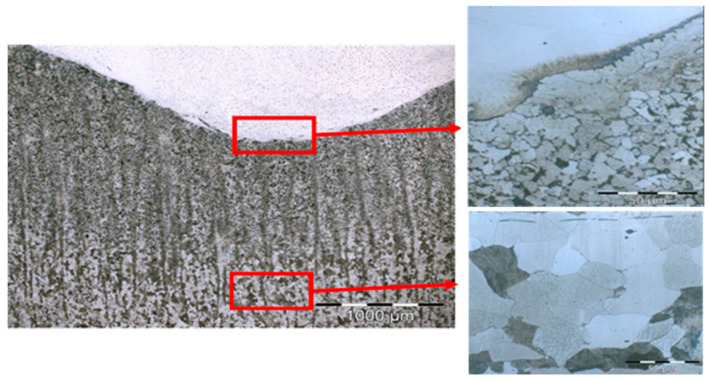
Micrographs of weld beads: Microstructure of the base material and heat affected zone. General view at 500× and detail of different zones at 1000×.

Figure 13 shows the metallograph results carried out on the weld bed corresponding to Test 5 with wire feeds of 4 and 5 m/min, with a travel speed of 400 mm/min, and with ×50, ×100, and ×500 magnification, made in the laboratory of Precisgal group and ENCOMAT group.

Figure 13 depicts the metallographic structure of the heat-affected zone in the transition area between stainless and carbon steel. Vilella’s reagent was used to reveal the microstructure of the samples. It consists of a ferritic–perlitic structure with lower contents of pearlite in the vicinity of the welded area. This can be explained by a certain decarburization of the area due to the lower carbon content of the stainless steel. Figure 13 shows the grain refinement in the heat-affected zone due to a recrystallization of the previously laminated structure induced by the welding process. As a consequence, an increase in hardness was detected in this area during the micro-hardness test. The grain size of the substrate undergoes a substantial modification in the heat-affected zone (HAZ). The grains closest to the root of the weld range in size from 10 to 20 microns. Following this, a recrystallized zone can be observed, with grains smaller than 5 microns. This represents a tenfold reduction in grain size compared to the base material, as shown in Figure 13, where the grain size is higher than 50 microns.

**Figure 14:**
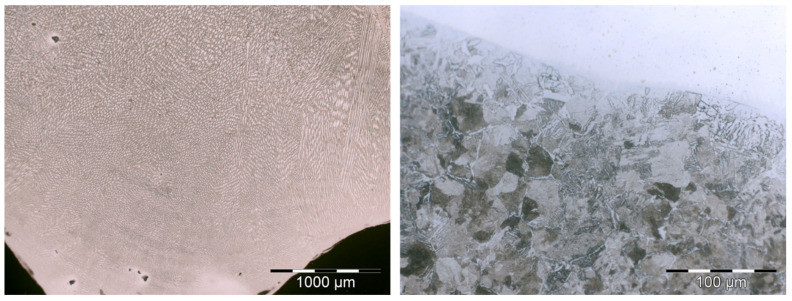
Microstructure of the austenitic weld microstructure (**right**) and the acicular microstructure detected in the interphase (**left**).

Figure 14 depicts the typical columnar austenitic grain microstructure associated with arc welding processes that was observed. Within the austenite (γ) matrix, represented in white, ferrite (δ) is dispersed, as depicted in gray following the columnar grain borders. Adjacent to the fusion line, grains exhibit nearly vertical growth. Eventually, the fine columnar structures transform into coarse columnar structures, with secondary dendrites becoming increasingly prominent at greater distances from the fusion line [35]. Several authors reported on the formation of hard phases [36], as bainitic structures or Widmästaten ferrite in the HAZ of clad deposits is the main reason for the hardness increase in the interphase between the welds and the base mild steel. This kind of acicular structure can be appreciated in Figure 14.

Table 5 shows the results obtained after the Vickers hardness test in (P1_5.1) (as well as the results in HRc) at different points on the weld bead (P1_5.1 see Table 3). Hardness measures from point 1 to 10 correspond to 316LSI material. Measures from point 11 to 14 correspond to the heat affected zone (HAZ). Measures from point 15 to 21 correspond to S275JR.

**Figure 15:**
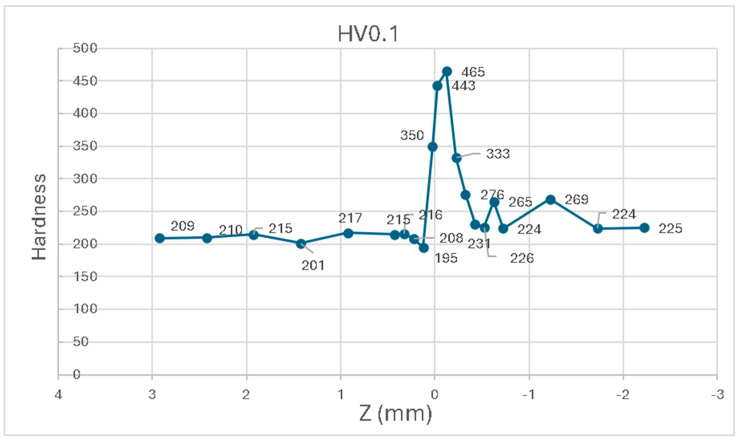
Hardness of HV0.1 versus z (mm).

Figure 15 graphically presents the results, where the incremental hardness of the 0.03 mm tempered zone of filler 316LSI and the HAZ zone of about 0.33 mm can clearly be observed. Despite the average carbon content being ≤0.2% and the differing hardnesses, good interlayer adhesion is achieved. There is also an unhardened zone (−0.4 mm below the weld line), in which there is hardly any change in the degree of hardness of S275JR between HV0.1, 224, and 276.

Andrade et al. [37] have obtained similar results related to stainless steel 316, which coincide with the equivalent zones of 316LSI. Similar results for hardness are also found in the work of Chen et al., at 100% 316LSI [38]. However, the microhardness results of Motwani et al. differ when it comes to 316LSI stainless steel, being between 160 and 190, slightly lower than those studied outside the HAZ zone, which are between 195 and 217 [39].

The results of Masek et al. [40], who compared the relative machinability of rolled AISI316L with that obtained by WAAM and LPC (Laser powder cladding), showed a different chemical composition and structure of the material with increased values of cutting forces and voids in the surface profile, after machining of the printed specimens.

The way a material solidifies affects how well the metal material can be deposited in 3D, as well as the characteristics it exhibits after deposition. This process is often understood by looking at the initial stages of solidification, how they change over time and what compositions they give rise to. According to the Schaeffler diagram, the solidification mode of stainless steel 316 is primarily austenite, with ferrite solidified secondarily from the melt (AF) [41].

**Figure 16:**
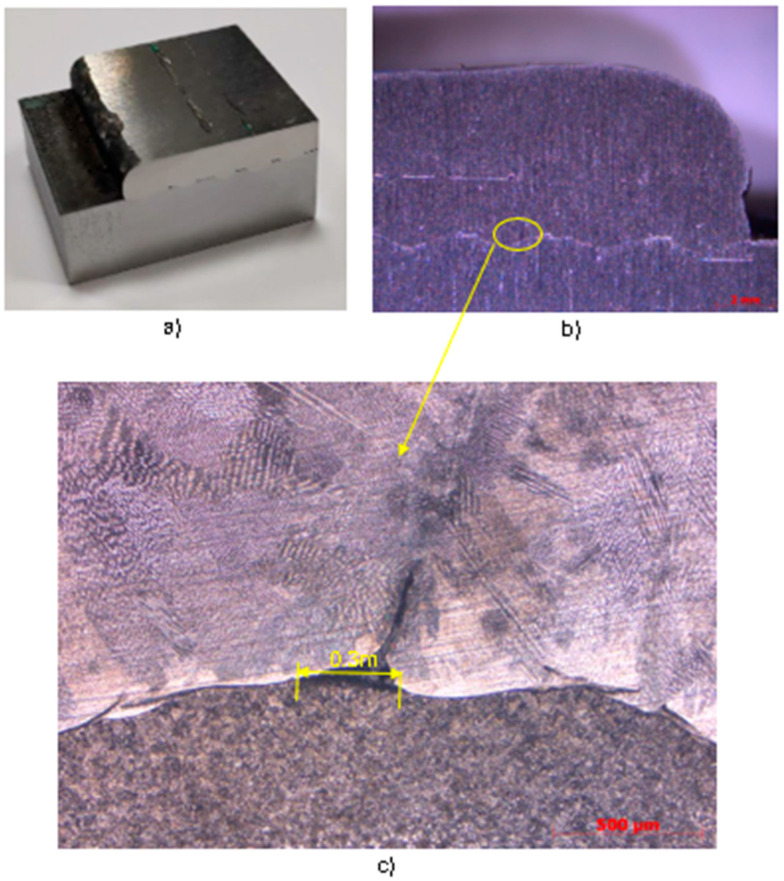
(**a**) Section of experiment C5 with *wf* = 4m/min; (**b**) macro-photograph of experiment C5 with linear disposition and intermediate machining; (**c**) micrograph of contact zone 316LSI and S275JR.

Figure 16a shows the section of the machined sample of experiment C5 with the intermediate machining layer before the second layer. This section shows the appearance of micro pores between the interstitial layer of the 316LSI over the S275JR. Figure 16b shows the macro photography of the section of experiment C5-7, with a wire speed equal to 4 m/min, 15 horizontal layers, and 2 vertical layers with intermediate machining, made with a ZEISS STEMI 305 trinocular stereomicroscope. In the image, some pores can be seen in both the first and second layers, in which linear discontinuities can also be seen, corresponding to the overlapping on the facing layer. These pores in experiment C5 are 70% smaller than the pores found in experiments C2 and C3, resulting from machining of the stress–strain specimens.

Figure 16c shows the detail of the Vilella reagent etched metallography, where the pore is visible for 0.3 mm of its max length. On the other hand, the austenitic and ferritic structures can be seen more clearly.

### 3.3. Tensile Test Results

Four tensile tests were carried out. The selected specimens were obtained without the appearance of external pores in the experiments of horizontal samples (C2, and C3). Dimensions of width (*wa*) greater than 15 mm and height greater than 3 mm were considered. The milled (M3 and M4) and electro discharge machining (EDM) specimens were produced with a thickness of 5 mm and 3 mm, respectively.

**Figure 17:**
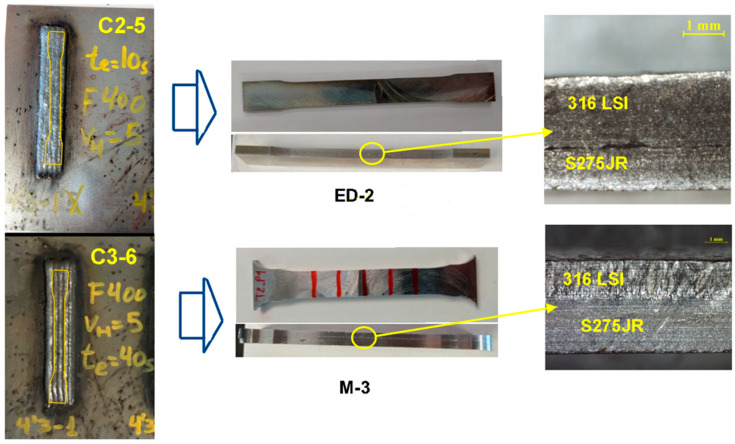
Machining of tensile samples and details of section of ED-2 and M-3.

Figure 17 shows the experimental samples of horizontal beads C2-5 and C3-6, their final tensile samples, and details on the union of the two materials. The dividing line of the two materials can be observed in the case of electro discharge machining tensile sample ED-2.

**Figure 18:**
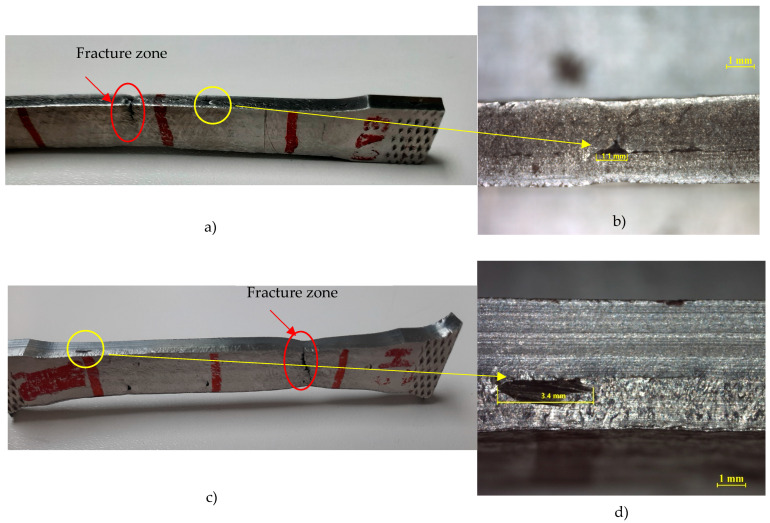
Results tensile test: (**a**) ED-2 test with fracture zone; (**b**) Measuring pore ED-2 with fracture zone; (**c**) M-3 test; (**d**) Measuring pore M-3.

Some pores can also be observed in the milling machining M-3 and ED-1 tension specimens. Porosity is a natural phenomenon associated with surfacing and using additive technologies, although it is not a dominant element. The focus of this article is on the behaviour of the bimetallic structure, and the porosity that arises from the use of these technologies was made in such a way that it has relatively little influence. It is confirmed from Figure 18 that the constriction and fracture zone of the sample occurs in areas where no visible macro pores are detected. This is because the pore sizes were not large enough to significantly affect mechanical properties. The authors are fully aware of this phenomenon; hence it is analysed by means of computed tomography with software that allows both the pore size and their spatial distribution to be assessed. An example of such an analysis for WAAM surfaces is presented in publication [30]. Generally, reducing the heat input can improve the microstructure and reduce the porosity. Regarding the average pore diameter in the case of the AA5356, the higher the travel speed, the lower the pore diameter. As the cooling time increases, the size of the pore diminishes. The optimum setup must include higher travel speeds and longer cooling times to reduce pore diameter. Thus, the size of the pores gets smaller when heat input and heat accumulation are lower.

Figure 18 shows in (a) and (c) the machined samples of EDM-2 and M3 where the fracture zones and visual macro pores can be observed.

Figure 18b,c presents details of the visual macro pores and, as can be noted before, there is no coincidence between the fracture zone and visible macro pores. These macro pores probably happened due to a fault in continuity of the welding process. The measurements of the max length of the pores are 1.1 mm in the case of ED-2, and 3.4 mm in the case of M3. The appearance of macropores provides justification for the experiments in which the intermediate machining of layers, experiments C5, is carried out in order to avoid macropores, homogenise the layers, improve the deposition, and allow it to be treated by CT computer tomography to study the porosity as a future research study. The results of Dash et al. [42] on ER70S steel decrease macropores, or incomplete fusion, by 99% by applying actively cooling deposition. This procedure is interesting as an inclusion into future research.

The results of the tensile test of the samples are shown in Table 6.

The results of the yield stress of the samples are 50% higher than the substrate S275JR, according to Kelly et al. [43]. As for the results of yield stress compared to those of 316LSI, it is also found that they are superior by 43%. In future work, it would be beneficial to consider subsequent austenitisation and normalisation heat treatments after WAAM, in accordance with the findings of Kabaldin et al. [44], which have demonstrated the potential for achieving 40% increases in bimetallic material (ER70 + ER309) tensile strength.

## 4. Conclusions

This research study about the characterization of a bimaterial consisting of stainless steel 316LSI on construction steel S275JR, using a manufacturing hybrid method (CMT-WAAM, and machining) draws the following conclusions:

- A methodological investigation has been carried out starting with welding beads of 316LSI on a S275JR plate, followed by overlapping five beads and conducting final experiments with several vertical layers.
- Optimal bead conditions have been established for wire speeds of 4 m/min and 5 m/min, and a process speed of 400 mm/min.
- The results of the overlap experiments show that the best deposition results are achieved with an overlap of 1 mm over an average bead width of 3.3 mm, i.e., a 28% overlap.
- It is noted that cooling time does not significantly influence the final geometry of the coatings.
- Regarding the path strategy, the Go&Forth technique is selected, with a cooling time of 10 s.
- In coating tests, the crossing factor of trajectories does not have a major influence on the dimensional behavior of the beads.
- Regarding metallographic analysis, the filler material presents an austenitic columnar structure. In the base material, a bainitic structure, inferred via grain refinement, was detected in the heat-affected zone (HAZ).
- An increase in hardness is observed in the heat-affected zone.
- When analyzing the two materials in the welding pair, the standard for S275 steel does not specify a grain size for the material; however, steels with a fine grain have a higher tensile strength, greater ductility, and are less distorted during welding. The solution of using low-energy welding, among other advantages, produces a lower overall alteration of the grain size in the HAZ, which favors a lower distortion of the grain size outside the fusion pool.
- Regarding austenitic stainless steel, grain size properties mainly affect its creep behaviour, so a larger grain size favours this property. However, here the authors are looking for corrosion protection, so a melt pool with a larger grain size would produce better corrosion behaviour, which is favoured, as explained in the introduction, by the use of cold-welding technology.
- In the results obtained from the tensile tests of the bimetallic material, an increase in mechanical strength and yield strength is observed in the tested specimens

As future work, the tomographic study of samples is proposed to observe the porosities of 316LSI coatings on S275JR and their influence on the mechanical properties of the bimetallic material. Additionally, it is also considered interesting as future work to establish if there is dilution of the alloying elements present in stainless steel 316LSI into carbon steel as a result of temperature.

While not the aim of this paper, extra corrosion protection, achieved through the use of welding stainless steel as a coating, could be observed. It is also known that welding filler materials usually have higher alloying to compensate for this loss during the welding process. The CMT process minimizes the loss of alloying components, a fact that increases the protection of the recharged area. The corrosion comparative between a laminated AISI316LSi and another one reached with WAAM technology coating S275 steel have been raised as a future line of study.

As in the case of new parts manufacturing, their repair by means of a coating with WAAM technology is proposed for the recovery and increase of the useful life of parts with high added value. In future studies, this could be considered not only in terms of protection against corrosion, but also for the reconstruction of areas with different properties depending on use cases.

## Figures and Tables

**Table 1:** Chemical composition of 316 L stainless steel wire (values in%).

C	Mn	Si	Ni	Cr	Mo	Cu	V	Co
0.03 max	1.99	0.65–1.0	11.52	16.55	2.90	0.17	0.08	0.56

**Table 2:** Chemical composition of S275JR structural steel plate (values in%).

C	Si	Mn	P	S	Ni	Cr	Mo	Cu	Al
0.08	0.22	0.57	0.025	0.017	0.14	0.1	0.02	0.5	0.002

**Table 4:** Dimensional results of different experiments.

Code	h_b	v_l	ov_l (mm)	p_st	*ct* (s)	Cross Layer	mch	*wf* (m/min)	*ha* (mm)	*hs* (mm)	*wa* (mm)	*ws* (mm)
C1_1	5	1	1.5	ZigZag			No	4	4.3	0.3	13.1	0.5
C1_2	5	1	1	ZigZag			No	4	3.9	0.3	15.0	0.6
C1_3	5	1	0.5	ZigZag			No	4	3.5	0.1	16.2	1.0
C1_4	5	1	0	ZigZag			No	4	3.4	0.1	17.1	0.8
C1_5	5	1	1.5	ZigZag			No	5	4.4	0.2	15.7	0.9
C1_6	5	1	1	ZigZag			No	5	4.0	0.3	17.4	0.1
C1_7	5	1	0.5	ZigZag			No	5	3.5	0.2	19.4	0.4
C1_8	5	1	0	ZigZag			No	5	3.3	0.1	21.1	0.7
C2_1	5	1	1.5	Go&Forth	10		No	4	4.4	0.5	13.7	0.8
C2_2	5	1	1	Go&Forth	10		No	4	3.9	0.4	15.5	0.6
C2_3	5	1	0.5	Go&Forth	10		No	4	3.5	0.2	16.6	1.5
C2_4	5	1	0	Go&Forth	10		No	4	3.4	0.4	19.5	0.9
C2_5	5	1	1.5	Go&Forth	10		No	5	4.7	0.4	16.9	0.8
C2_6	5	1	1	Go&Forth	10		No	5	4.0	0.4	18.3	0.3
C2_7	5	1	0.5	Go&Forth	10		No	5	3.3	0.1	20.4	0.4
C2_8	5	1	0	Go&Forth	10		No	5	3.3	0.3	23.0	0.6
C3_1	5	1	1.5	Go&Forth	40		No	4	3.7	0.3	14.4	0.6
C3_2	5	1	1	Go&Forth	40		No	4	3.9	0.1	15.3	0.7
C3_3	5	1	0.5	Go&Forth	40		No	4	2.9	0.4	16.9	0.3
C3_4	5	1	0	Go&Forth	40		No	4	3.0	0.2	19.2	0.4
C3_5	5	1	1.5	Go&Forth	40		No	5	4.2	0.1	16.5	0.7
C3_6	5	1	1	Go&Forth	40		No	5	3.6	0.3	18.7	0.7
C3_7	5	1	0.5	Go&Forth	40		No	5	3.0	0.1	20.2	0.5
C3_8	5	1	0	Go&Forth	40		No	5	3.4	0.2	22.3	0.4
C4_1	15	1	1	Go&Forth	10		No	5	3.9	0.4	55.0	1.0
C4_2	15	1	1	Go&Forth	10		No	5	4.0	0.3	55.0	1.0
C4_3	15	2	1	Go&Forth	10	Cr_layer	No	5	8.0	0.7	55.0	1.0
C4_4	15	2	1	Go&Forth	10	Same	No	5	7.9	0.6	55.1	1.0
C4_5	15	3	1	Go&Forth	10	Cr_layer	No	5	12.1	0.9	55.1	1.0
C4_6	15	3	1	Go&Forth	10	Same	No	5	11.8	1.0	55.1	1.0
C5_1	15	1	1	Go&Forth	10		No	5	3.9	0.4	55.0	1.1
C5_2	15	1	1	Go&Forth	10		No	5	4.0	0.3	55.0	1.0
C5_3	15	1	1	Go&Forth	10		No	4	3.9	0.4	46.4	1.7
C5_4	15	1	1	Go&Forth	10		No	4	3.9	0.4	46.3	1.7
C5_5	15	2	1	Go&Forth	10	Same	Yes	5	7.4	0.3	55.0	1.0
C5_6	15	2	1	Go&Forth	10	Same	Yes	5	7.3	0.3	55.0	1.1
C5_7	15	2	1	Go&Forth	10	Same	Yes	4	6.3	0.3	46.5	1.4
C5_8	15	2	1	Go&Forth	10	Same	Yes	4	6.3	0.3	46.5	1.4
C5_9	15	2	1	Go&Forth	10	Cr_layer	Yes	5	7.3	0.3	55.0	1.0
C5_10	15	2	1	Go&Forth	10	Cr_layer	Yes	5	7.3	0.3	55.0	1.0
C5_11	15	2	1	Go&Forth	10	Cr_layer	Yes	4	6.3	0.3	46.5	1.4
C5_12	15	2	1	Go&Forth	10	Cr_layer	Yes	4	6.3	0.3	46.3	1.7

**Table 5:** Results of hardness measurement in test.

Zone	Points	Z (mm)	HV0.1	HRc
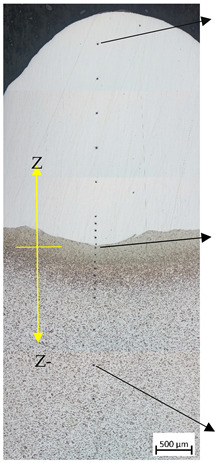	1	2.925	209	14.7
	2	2.425	210	14.3
	3	1.925	215	15.7
	4	1.425	201	13.1
	5	0.925	217	16.3
	6	0.425	215	15.8
	7	0.325	216	15.9
	8	0.225	208	14.5
	9	0.125	195	11.9
	10	0.025	350	35.5
	11	−0.025	443	44.7
	12	−0.125	465	46.5
	13	−0.225	333	33.6
	14	−0.325	276	26.6
	15	−0.425	231	18.6
	16	−0.525	226	17.8
	17	−0.625	265	24.8
	18	−0.725	224	17.5
	19	−1.225	269	25.4
	20	−1.725	224	17.5
	21	−2.225	225	17.6

**Table 6:** Results of tensile test of samples.

From Sample	Tension Sample	Thickness (mm)	Section (mm^2^)	Fmax (N)	Yield Stress (MPa)	Ultimate Stress (MPa)	Strain (%)
C2-7	ED_1	3.4	38.1	23,628.1	415.6	620.5	15.0
C2-5	ED_2	3.3	37.9	23,724.2	423.2	623.0	22.5
C3-6	M_3	4.9	55.9	33,827.2	416.6	604.6	22.5
C2-6	M_4	4.8	56.0	31,393.8	----	560.6	19.5

## Data Availability

The original contributions presented in the study are included in the article, further inquiries can be directed to the corresponding author.
